# Editorial: Emerging genomic technologies for agricultural biotechnology: current trends and future prospects

**DOI:** 10.3389/fpls.2023.1263289

**Published:** 2023-08-24

**Authors:** Md. Anowar Hossain, Hairul Azman Roslan

**Affiliations:** ^1^ Department of Biochemistry and Molecular Biology, University of Rajshahi, Rajshahi, Bangladesh; ^2^ Faculty of Resource Science and Technology, Universiti Malaysia Sarawak, Kota Samarahan, Sarawak, Malaysia

**Keywords:** CRISPR/Cas9, RNA-sequencing, quantitative trait loci, genome editing, marker assisted selection (MAS)

The current earth’s population of 7.6 billion is expected to reach 8.6 billion by 2030. The increased population will need more food than it can currently produce. However, world agriculture is facing severe challenges such as global climate change, exhausted resources, reduction of arable lands and various pathogens. Advances in genomic technologies may offer potential solutions to these agricultural problems. New genomic technologies such as, Next generation sequencing (NGS), Ribonucleic acid sequencing (RNA-Seq), Clustered Regularly Interspaced Short Palindromic Repeat-Cas9 (CRISPR/Cas9), Transcription activator-like effector nucleases (TALENS) and Oligonucleotide‐directed mutagenesis (ODM) as well as doubled haploids, molecular markers and mapping populations have been developed and utilized for increasing the crop production. Together with the rapidly expanding availability of genome sequence data, these technologies have the potential to transform plant breeding.

In this Research Topic we aim at the collection of articles on application of cutting-edge genomic technologies to improve various crops, vegetables and fruits which includes two reviews and seven original articles.

In first review, Aziz et al. explored the deployment of GM crops and their effects on sustainable food production systems which provided a comprehensive overview of the cultivation of GM crops and the issues preventing their widespread adoption, with appropriate strategies to overcome them. They also presented new tools for genome editing technology with special reference of CRISPR/Cas9 platform. They outlined the role of crops developed through CRISPR/Cas9 for sustainable development goals by 2030.

Nowadays in the post genomic era, transcriptomic or RNA-Seq technology focuses on functional studies of transcriptomes. It is a high-resolution, sensitive and high-throughput NGS approach used to study non-model plants and other organisms. RNA-Seq is an important technique for predictions and functional analysis of genes that improves gene ontology biological processes, molecular functions, and cellular components, but still now there is limited information available on this topic. Tyagi et al. nicely presented the recent genomics technologies such as first generation, second generation (Next generation) and third generation for functional genomic studies. Their review article focused currently used technology in details and forthcoming strategies for improving transcriptome sequencing technologies for identifying the functional of genes in various plants using RNA-Seq technology, based on the principles, development, and applications.

In the first article, O’Connell et al. used genome wide association studies and genomic selection to identify the Single Nucleotide Polymorphism (SNP) markers for red rot resistance. *Colletotrichum falcatum* is a causal agent of red rot disease of sugarcanes worldwide including India. They identified a single 14.6 Mb genomic region in Sorghum which in turned helps to map the Quantitative Trait Loci (QTLs). Finally genetic analysis nearby the SNP markers linked to the QTL revealed many biotic stress responsive genes within this QTLs, including a cluster of four chitinase A genes.

The primary mapping of QTLs is a molecular technique that has been widely used technique for the identification of candidate genomic regions controlling various crop traits based on different types of genetic markers. This article, Amanullah et al. performed the whole Genome Sequencing (WGS) for two watermelon varieties. They identified the 33 QTLs at different genetic positions across the eight chromosomes of watermelon for the ovary, fruit, and seed related phenotypes. Twenty-four QTLs were mapped as major-effect and 9 QTLs were identified as minor-effect QTLs across the flanking regions of Cleaved Amplified Polymorphic Sequences (CAPS) markers. We hoped that their detected QTLs provide critical genetic information concerning the watermelon phenotypes and fine genetic mapping could be followed to confirm them.

In the next article, Sultana et al. successfully identified 5,684 differentially genes expressed genes (DEGs) from transcriptome data that control the allelopathic interaction between banyardgrass and rice. Among them, 388 genes were transcription factors and associated with momilactone and phenolic acid biosynthesis which could play critical roles in allelopathy. Moreover, they identified the up-regulated and down-regulated genes at certain condition, which could be involved in the secondary metabolites’ biosynthesis and developmental processes; respectively. They also detected some common DEGs between rice and banyardgrass which suggested different mechanisms underlying allelopathic interaction in these two species. Authors believed that their study could provide a valuable genetic resource for rice and banyardgrass associated candidate allelopathy genes and should be useful for controlling weeds, which would outcome in the development of agriculture.

In this article, to overcome the variable efficiency of the guide RNA (gRNA) in the CRISPR/Cas mediated genome editing system for the improvement of agronomic traits of many crop plant species, Wang et al. used the *Agrobacterium*-mediated transient assays to evaluate the effectiveness of gRNAs for editing genes in tobacco and soybean. They established a new transient assay system to validate the effectiveness of gRNAs before generating stable transgenic plants.

Trypsin inhibitor (TI) present in the soybean seeds is an anti-nutritional factor that hampers the digestibility of soybean meal in the human intestine tract. To develop the low TI soybeans, in their next article, Wang et al. identified two seed-specific TI genes such as KTI1 and KTI3. They developed TI-mutant lines of soybeans through CRISPR/Cas9 mediated genome editing approach and also detected co-dominant selection markers for mutant alleles. Authors expected that the kti1/3 mutant soybean line and associated selection markers would help in accelerating the introduction of low TI trait into elite soybean cultivars in the future.

To discover the heat stress responsive genes of maize, Jagtap et al. employed the transcriptomic studies of two inbred lines, M 11 (sensitive to heat shock) and CML 25 (tolerant to heat shock), under intense heat stress at 42°C during reproductive stage from flag leaf, tassel, and ovule. They identified 1127 up-regulated and 1037 down-regulated differentially expressed genes (DEGs) with 1151, 451, and 562 DEGs in comparisons of LM 11 and CML 25, corresponding to a leaf, pollen, and ovule, respectively. Functional analysis revealed that DEGs associated with transcription factors (TFs), heat shock proteins (HSP20, HSP70, and HSP101/ClpB), as well as genes related to photosynthesis (PsaD & PsaN), antioxidation (APX and CAT) and polyamines (Spd and Spm). Their gene expression analysis found higher expression of HS-responsive genes in CML 25, which might explain why CML 25 is more heat tolerant in heat stress.

Due to the large genome size, hexaploidy nature and high percentage of repetitive regions of wheat, it is difficult to map yield-related QTLs. Ma et al. conducted high-density SNP arrays technology to construct genetic map and identify twelve environmentally stable QTLs for yield-related traits in wheat. Among them QTkw-1B.2 and QPh-4B.1 could be novel QTLs which could be used for wheat breeding programs. We hope that researchers and scientists working on crop improvement for sustainable food production will be benefited from this Research Topic in future.

The articles in this Research Topic provide a fascinating overview of the latest genomic technologies for agricultural biotechnology. These technologies, described in the articles, provide novel solutions to global agriculture concerns such as climate change, resource scarcity, and pathogen outbreaks. The papers discussed a variety of genomic technology applications, from developing GM crops to identifying QTLs and genes that respond to heat stress in different crops. The findings can help improve crop output, build sustainable food systems, and increase the nutritional content of agricultural products. We can expect significant advancements in plant breeding and agricultural practices as genomic technologies continue to advance and researchers and scientists collaborate, ultimately contributing to global food security and the achievement of sustainable development goals.

## Author contributions

MH: Conceptualization, Data curation, Formal Analysis, Investigation, Supervision, Writing – original draft. HR: Writing – review & editing.

